# The Future of mRNA Vaccines: Potential Beyond COVID-19

**DOI:** 10.7759/cureus.84529

**Published:** 2025-05-21

**Authors:** Somya Saxena, Vineet Mandrah, Wali Tariq, Papri Das, Kumar Sambhav, Salam Himika Devi

**Affiliations:** 1 Internal Medicine, KD Medical College, Mathura, IND; 2 General Surgery, Chhindwara Institute of Medical Sciences, Chhindwara, IND; 3 Forensic Medicine, Krishna Mohan Medical College, Mathura, IND; 4 Community Health Nursing, Brainware University, Kolkata, IND; 5 Anatomy, All India Institute of Medical Sciences, Bilaspur, Bilaspur, IND; 6 Life Sciences, Manipur University, Canchipur, IND

**Keywords:** autoimmune diseases, cancer immunotherapy, covid-19, genetic disorders, mrna therapeutics

## Abstract

In recent years, mRNA therapeutics have emerged as a promising platform in treating a wide range of diseases, including cancers, infections, genetic disorders, and autoimmune diseases. This review focuses on the clinical impact of mRNA-based treatments and their transformative potential in modern medicine. mRNA therapeutics utilize the host's cellular machinery to produce target proteins, enabling highly specific and customizable treatments. In the case of cancer, mRNA vaccines to stimulate immune responses against such tumor-specific antigens are being developed in a personalized manner. Infectious diseases are also an indication for which mRNA vaccines have shown a significant effect on preventing viral infection, as the global success of mRNA COVID vaccines demonstrates. Among genetic disorders, mRNA therapy presents a new way to restore defective proteins and reverse the underlying pattern of genetic defects. Furthermore, mRNA treatment of autoimmune diseases aims to generate immune tolerance and avoid traditional immunosuppressive therapy. Advances are being driven by the discovery of new technologies to stabilize, deliver, and modulate the immune system around mRNAs. Recent advances in delivery systems and RNA stabilization have further expanded the potential of mRNA vaccines for viral diseases. mRNA therapeutics have the advantage of being rapidly developed and adaptable to a wide range. Research on further improvement of the delivery mechanisms and long-term safety will be necessary for extending the clinical applications of mRNA therapeutics.

## Introduction and background

Background of mRNA vaccines

The origins of mRNA vaccines lie in the 1990s when researchers in search of more innovative approaches to combating disease turned to gene vaccines. Early exploration of mRNA technology faced challenges due to the molecule's instability. However, by the early 2000s, significant advancements were made, most notably the development of lipid nanoparticles (LNPs), which protect mRNA from degradation and facilitate its delivery into cells. These innovations laid the foundation for the success of mRNA vaccines [1], particularly during the COVID-19 pandemic.

Successful mRNA vaccines for COVID-19

As the first time mRNA vaccines were rolled out collectively, Pfizer-BioNTech (BNT162b2) and Moderna (mRNA-1273) were among the first to be given Emergency Use Authorization (EUA) recognition by agencies like the U.S. Food and Drug Administration (FDA). Extensive phase 3 trials [2,3] showed that each vaccine had more than 90% efficacy in preventing symptomatic COVID-19 infection.

**Figure 1:**
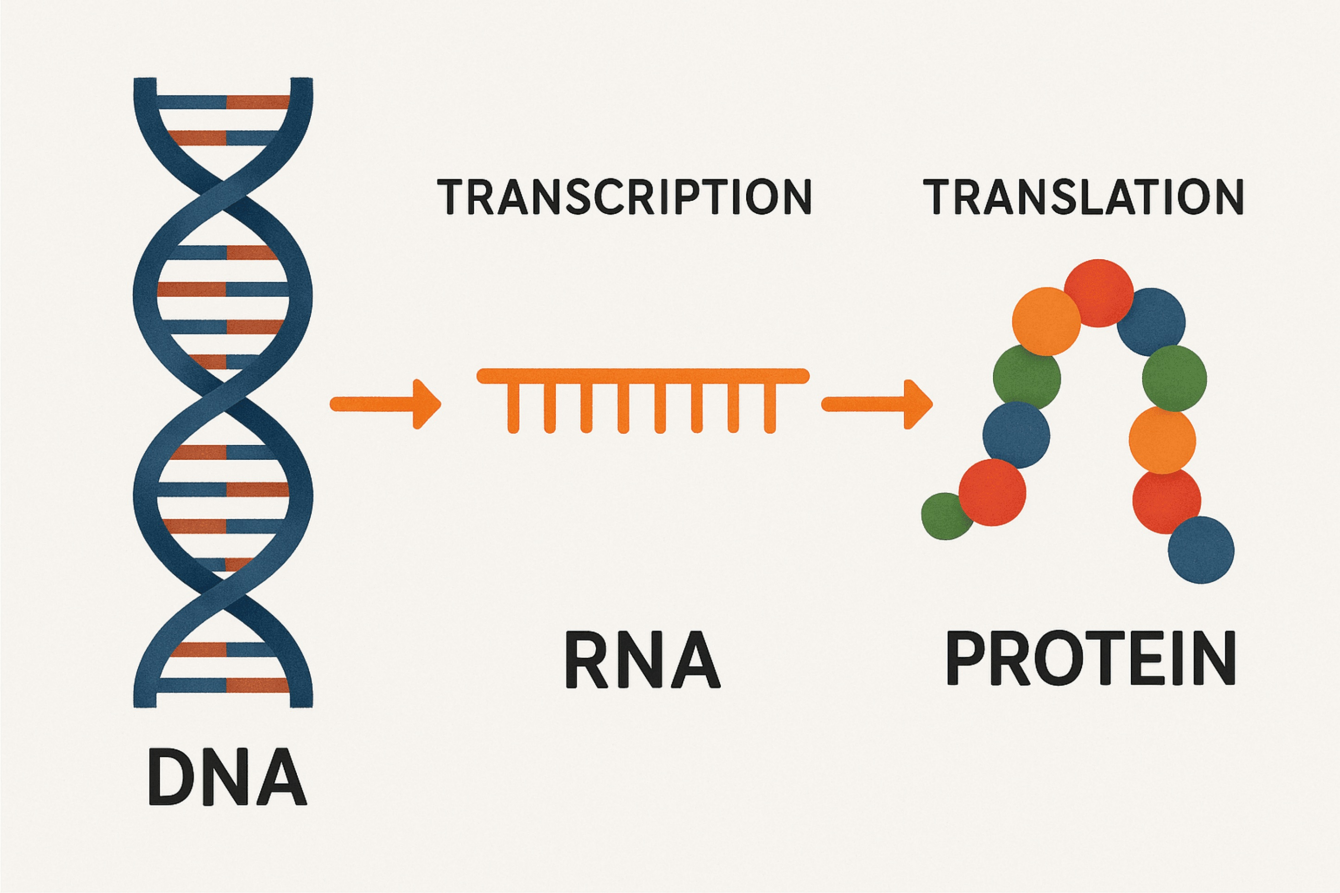
Schematic representation of the central dogma Created by the authors using DALL-E 3 software

Figure 1 illustrates the fundamental process by which genetic information is expressed within cells. DNA, the hereditary material, is transcribed into mRNA, which serves as a temporary copy of the gene. The mRNA then undergoes translation by the cellular machinery to synthesize specific proteins. These proteins perform essential structural and functional roles in the cell and are the basis of many mRNA-based therapeutic strategies.

Overview of the importance of mRNA vaccines in public health

The swift development of mRNA vaccines in the COVID-19 pandemic has shown their capability to tackle emerging infectious disease threats. In contrast to conventional vaccine approaches that tend to take years to shift from design to production, mRNA platforms permit more rapid initial development. But these benefits need to be recognized as being subject to logistical and regulatory hurdles such as cold-chain storage needs, scalability of manufacture, and requiring efficient approval mechanisms [4]. In addition, mRNA vaccines have been found to have a favorable safety profile and provide flexibility to adjust to mutating viral strains [5].

Purpose of the review

The purpose of this review is to explore the potential of mRNA vaccines beyond COVID-19. It aims to examine emerging research, applications, and possible advancements in the field of mRNA vaccine technology, including its use for cancer, autoimmune diseases, and other infectious diseases.

## Review

Mechanism of action: how mRNA vaccines work

Fundamentals of mRNA Technology

Basic Principles of mRNA Vaccine Function: The mechanism behind Messenger RNA (mRNA) vaccines is to take advantage of a synthetic mRNA sequence encoding a viral antigen to cue human cells into producing a protein related to the virus, thus triggering an immune response. This method reflects an essential deviation from typical vaccinations that usually depend on either live attenuated or inactivated viral forms to stimulate protective immunity. After the delivery of mRNA, it goes into the cells where ribosomes interpret the mRNA sequence into a viral protein, commonly the spike protein for the SARS-CoV-2 virus in vaccines aimed at COVID-19, including those developed by Pfizer-BioNTech and Moderna. The translated protein is non-infectious but serves as an immunogen that activates the adaptive immune system. The antigen is recognized as foreign by the immune system, leading to activation of B-cells and T-cells that mediate both humoral and cellular immunity. These immune responses get the body ready to quickly respond to the actual virus if it is encountered in the future [6].

Evaluation of Traditional Vaccine Technologies in Comparison: Traditional vaccines typically fall into two main categories: vaccines that are live attenuated and vaccines that are inactivated. Live attenuated vaccines contain a virus able to multiply in the body, but it does not trigger sickness. The measles, mumps, and rubella (MMR) vaccine is a single illustration, along with the varicella (chickenpox) vaccine. Unlike live virus vaccines, on the contrary, inactivated vaccines include completely inactivated virus particles that cannot multiply inside the body. The polio vaccine, along with the hepatitis A vaccine, serves as an example.

**Figure 2:**
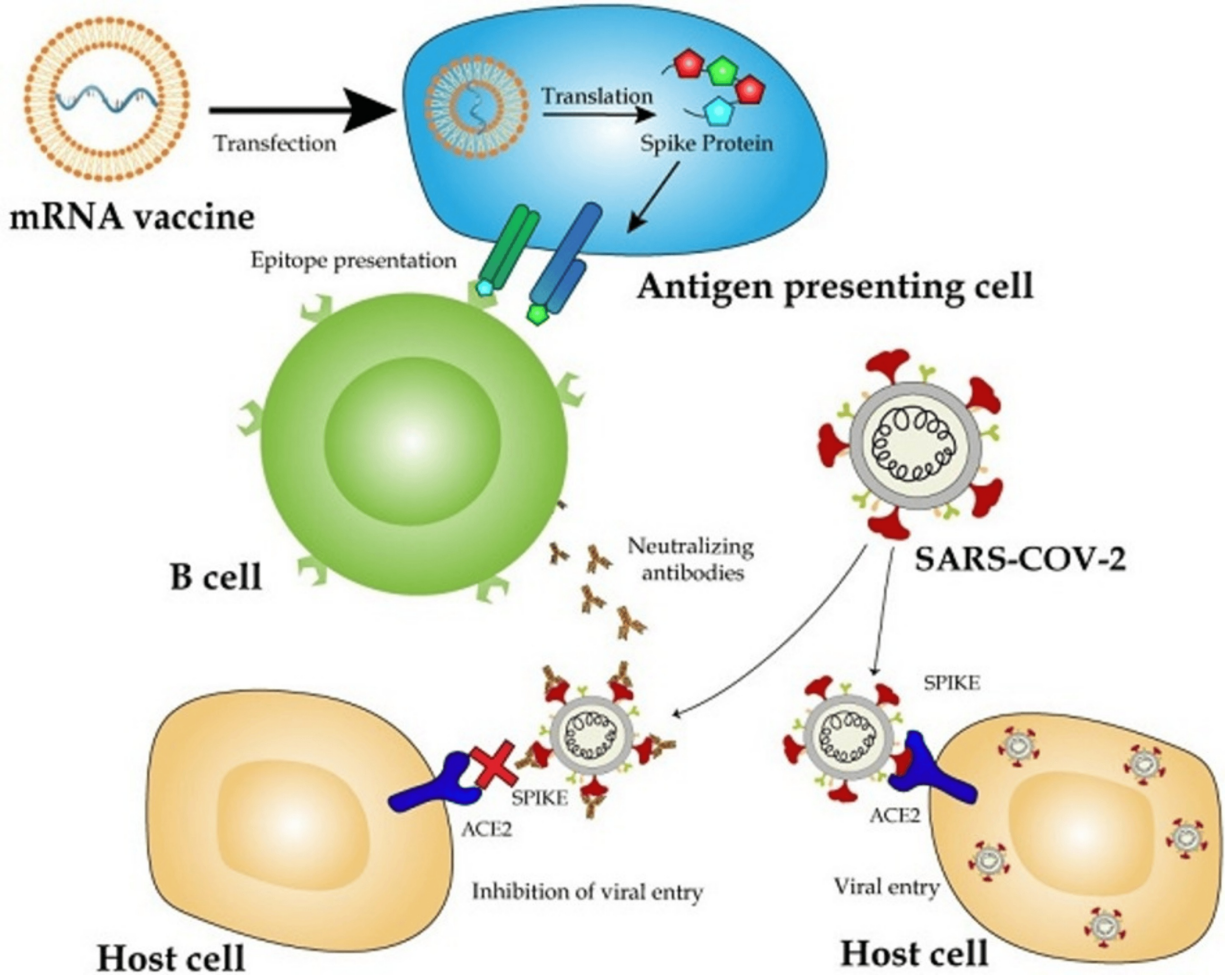
Working of mRNA Vaccine in Human Body Source: Park JW, Lagniton PNP, Liu Y, et al. mRNA vaccines for COVID-19: what, why and how. Int J Biol Sci. 2021;17(6):1446-1460. 10.7150/ijbs.59233 [7]. This figure is licensed under Creative Commons Attribution License (https://creativecommons.org/licenses/by/4.0/). ACE2: angiotensic-converting enzyme

Figure 2 shows how mRNA vaccines function against SARS-CoV-2. The mRNA is introduced into antigen-presenting cells (APCs), where it gets translated into the viral spike protein. The expressed protein is subsequently processed and presented through major histocompatibility complex (MHC) pathways: peptides are loaded onto MHC class I molecules, which stimulates CD8+ cytotoxic T cells, whereas presentation through MHC class II molecules stimulates CD4+ helper T cells. This dual pathway activates both cellular and humoral immune responses. In particular, B cells are activated to generate neutralizing antibodies against the viral spike protein and prevent its interaction with angiotensin-converting enzyme 2 (ACE2) receptors on host cells to block viral entry. These immune mechanisms act synergistically to clear infected cells and provide immunity against future infection [7].

Where traditional vaccines have shown promise, they also come with obstacles like longer development periods, the obligation for laboratory pathogen culturing, and possible risks for those with immune system disorders, when live attenuated vaccines are on hand. In opposition, mRNA vaccines can be rapidly synthesized in a laboratory, do not involve the handling of live viruses, and are likely safer for a broader population due to their exact targeted action [8].

Immunological response triggered by mRNA vaccines

The Role of mRNA in Protein Synthesis and Immune Activation

When a vaccine's mRNA enters cells, it does not fuse with the host's DNA but remains in the cytoplasm, where it is translated into protein by the cell’s ribosomes. For example, the mRNA used in the Pfizer-BioNTech and Moderna COVID-19 vaccines encodes the spike (S) protein of the SARS-CoV-2 virus, which is recognized by the immune system as a foreign antigen. Once synthesized, the spike protein is processed and presented on the cell surface via MHC molecules, making it detectable to immune cells. In the humoral response, B-cells are activated to produce antibodies that neutralize the spike protein, thereby preventing the virus from infecting host cells during future encounters. This mechanism is central to vaccine-mediated protection and can be rapidly adapted for other viral pathogens. Concurrently, the cellular response involves T-cells, particularly cytotoxic T-cells, which recognize and eliminate cells displaying the spike protein, thereby assisting in infection clearance and contributing to long-term immunity.

**Figure 3:**
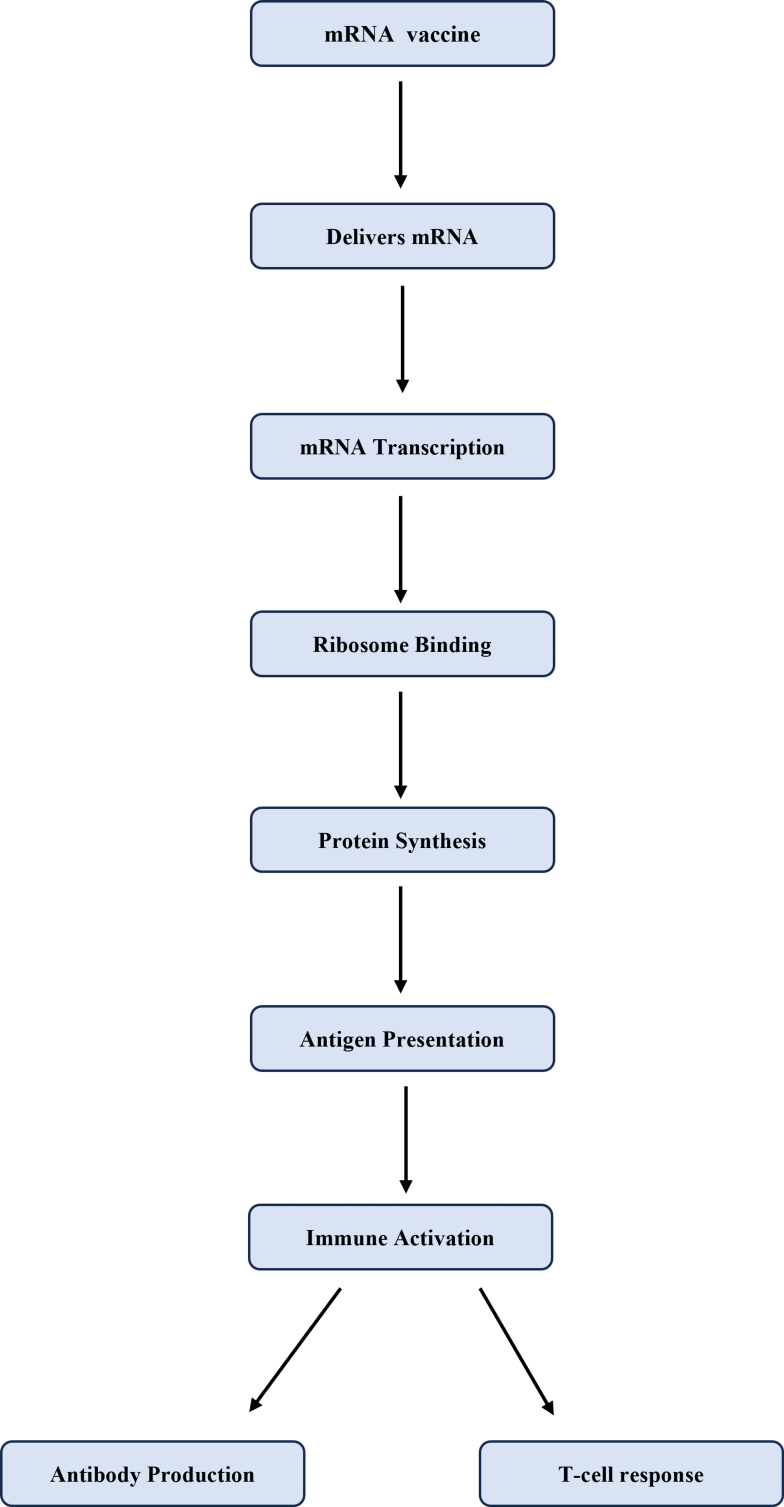
Role of mRNA in Protein Synthesis and Immune Activation Created by: Somya Saxena

Figure 3 conveys that this dual response is important for achieving effective protection against viruses, and mRNA vaccines are especially effective in stimulating these responses because of the strong antigen expression they generate [9].

The Relationship Between Safety Profile and Immune System Interaction

The principal anxiety related to all new vaccine technologies is their safety. mRNA vaccines have shown an encouraging safety profile as they lack live elements and cannot modify the host’s genetic makeup. The mRNA breaks down by itself in the body within a few days of injection, which lowers the chance of enduring side effects. The mRNA vaccines designed for COVID-19 showed mild to moderate side effects closely related to vaccines during clinical trials, including shot site pain, exhaustion, headache, and a mild spike in temperature. Rare serious effects, including anaphylaxis, have happened at a rate of about two to five cases per million doses given [10]. These responses are largely because of the lipid nanoparticles used for mRNA delivery, not the mRNA itself.

In addition, mRNA vaccines are devoid of infectivity and do not utilize any infectious agent, thereby diminishing the chance of both causing the disease they are designed to protect against, a potential problem with live attenuated vaccines. The other contribution of mRNA is that it permits the fast tailoring of vaccines to newly emerging variants, an ability that will be fundamental for addressing future pandemics and new viruses [11]. Delivering rapid development times, top efficacy, and an effective safety profile, mRNA vaccines constitute a landmark development in the field of vaccinology. Through its robust immune response stimulation via protein synthesis and its non-infectious properties, the technology establishes itself as a platform with great promise in preventing infectious diseases and also in therapeutic fields like cancer immunotherapy and chronic disease management. Although traditional vaccines have served us well, mRNA vaccines present a flexible and strong new resource for responding to both present and future public health challenges.

Beyond COVID-19: emerging applications of mRNA vaccines

Cancer Vaccines

Current Research on mRNA Vaccines for Personalized Cancer Immunotherapy: mRNA vaccines have demonstrated their ability to stimulate the immune system to go after and eliminate cancer cells in cancer immunotherapy.

**Figure 4:**
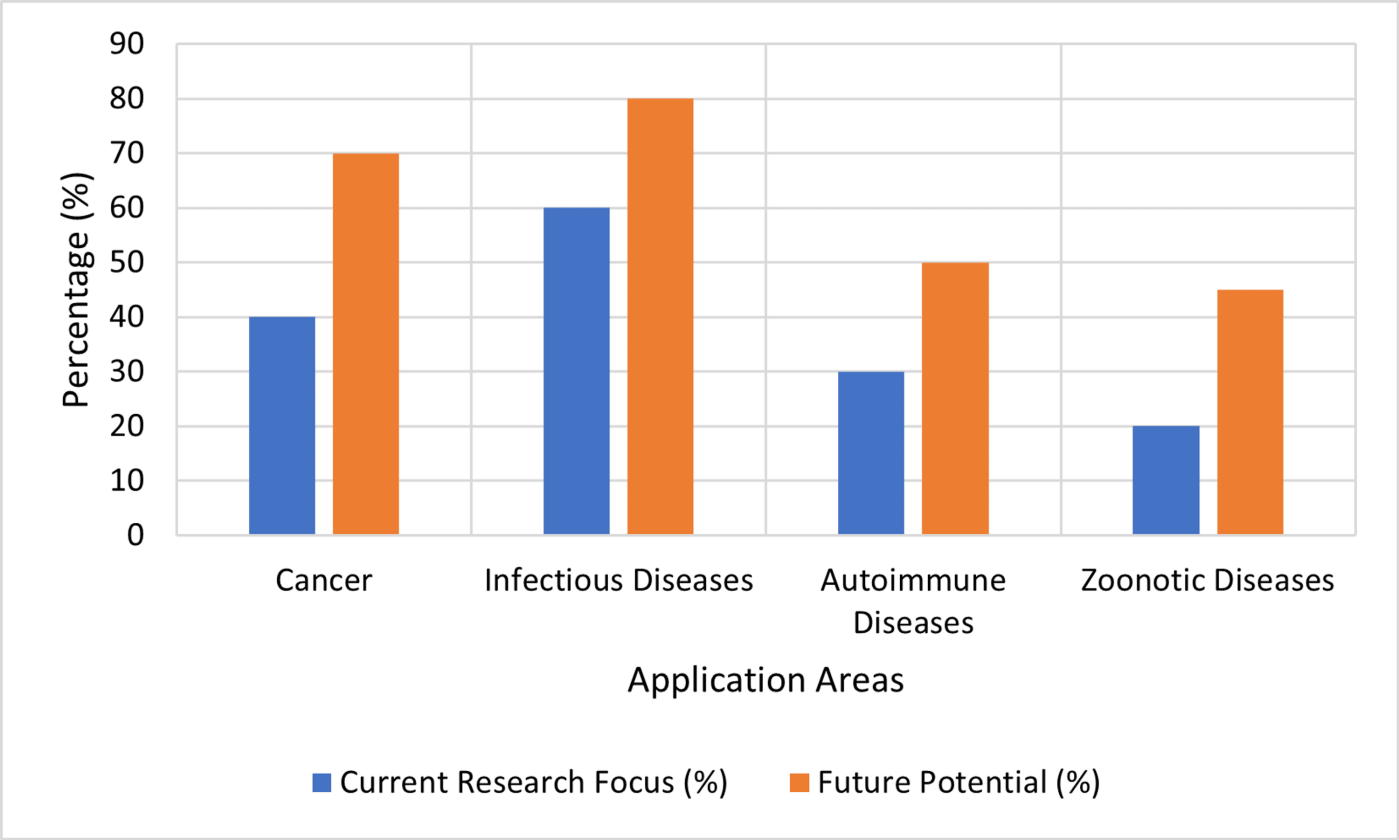
Current Research and Future Potential of mRNA Vaccines in Various Applications This figure has been created by the authors using data from references [1], [5], and [10].

Figure 4 charts the current research focus and future horizons of mRNA vaccines in different medical areas, including cancer, infectious diseases, autoimmune diseases, and zoonotic diseases. The blue bars illustrate the fraction of current research investment in each area, and the green bars reflect the likely future potential for mRNA vaccines in these sectors. Importantly, infectious diseases are in the lead regarding both current research attention (60%) and future possibilities (80%). At present, autoimmune diseases and zoonotic diseases are not getting much attention, but they have a strong potential for future growth (50% and 45%, respectively).

In contrast to infectious disease vaccines, cancer vaccines require the targeting of antigens specific to the tumour. mRNA vaccines facilitate the provision of tailored antigens based on the special mutations present in a person's tumour (neoantigens). Melanoma, lung cancer, and colorectal cancer are the subjects of intense study for personalized cancer mRNA vaccines. This personalized strategy enables the immune system to distinguish tumor-specific neoantigens and mount a targeted cytotoxic response against malignant cells [12]. However, tumor heterogeneity, antigen escape, and the immunosuppressive tumor microenvironment remain key obstacles to effective mRNA-based cancer immunotherapy [8].

Examples of Clinical Trials Currently Ongoing and Their Findings: Important research into an mRNA cancer vaccine involves the melanoma vaccine developed by BioNTech, which has delivered promising results in its early clinical trials. The results from the phase I trial of BNT111, an mRNA cancer vaccine, showed a strong immune response in patients with advanced melanoma. Moderna's mRNA-4157 is another illustration that is now in clinical trials for individuals with solid tumours. Findings early in the studies indicate that when combined with immune checkpoint inhibitors, the vaccine could result in an improved outcome for patients [13].

Challenges and Opportunities in the Development of mRNA Cancer Vaccines: The main challenges in developing mRNA cancer vaccines are to identify appropriate antigens for a variety of cancers and to ensure that the immune responses induced by the vaccine are directed against tumour cells. The challenges also include working in the immunosuppressive setting that tumours create. Nevertheless, this potential for opportunities is still translated in the virtue of the faculty of the mRNA technology to rapidly craft individualized vaccines and combine them with existing such as immune checkpoint inhibitors. Immune response durability and maintained efficacy are further to be analyzed [14].

Influenza and Other Respiratory Viruses

mRNA Vaccine Research for Influenza: Traditional influenza vaccines use inactivated or live attenuated virus strains to create their products, but they need annual updates according to predicted circulating strains. The manufacturing process requires an extended time, while mismatches between vaccine strains and circulating viruses reduce its effectiveness. The development of influenza vaccines through mRNA technology provides a faster approach that allows better adaptation to vaccine production. The production process of mRNA vaccines for influenza takes less time, which reduces the chance of mismatch with circulating strains. Moderna and other drug manufacturers develop mRNA vaccines for influenza, which demonstrate promising findings during their initial preclinical investigations [15].

Potential for Addressing Other Respiratory Viruses: Existing research indicates that mRNA vaccines could protect people from respiratory viruses, especially respiratory syncytial virus (RSV) and parainfluenza viruses, leading to major illness and death, mostly among young children and older adults. The research team at Moderna conducts clinical trials for RSV mRNA vaccines, which show promising results according to early laboratory findings [16].

Zoonotic Diseases and Emerging Infections

Potential Applications in Addressing Zoonotic Diseases: Zoonotic diseases, which move from animals to humans, threaten worldwide health as these viruses have caused outbreaks of Zika and both Ebola and Nipah. Research on mRNA vaccines continues because they provide a fast reaction system for emerging infectious diseases, due to their straightforward development and adaptable nature. The preclinical testing of Zika virus mRNA vaccines proved successful because they created protective immune responses. Equally important to other mRNA vaccine developments are ongoing efforts to create vaccines against the Ebola virus and the Nipah virus, with an emphasis on enhanced outbreak response against conventional vaccine manufacturing processes [17].

Opportunities for mRNA Vaccines Regarding Diseases Including Zika, Ebola, and Nipah Viruses: The World Health Organization has designated Zika virus, Ebola virus, and Nipah virus as the top priority diseases for vaccination. The quick design and manufacturing process of mRNA vaccines enables multiple benefits during outbreak situations. CureVac and Moderna have started research into mRNA vaccines for these viruses, obtaining encouraging findings from preclinical and early-phase clinical trials. Research has demonstrated that mRNA-1893 from Moderna successfully creates protective immune responses in animal test subjects [18].

Autoimmune and Chronic Diseases

Exploration of mRNA Technology to Treat or Prevent Autoimmune Diseases: Researchers conduct studies to determine mRNA vaccine potential for autoimmune disease protection and treatment, specifically for multiple sclerosis (MS) and rheumatoid arthritis (RA). The immune system's strength does not suffer from mRNA vaccines, unlike traditional treatments, because they offer the potential to control immune responses in a precise manner to minimize autoimmune damage without compromising holistic immune system operations. Findings from research conducted ahead of clinical trials reveal that mRNA vaccines can create tolerance toward self-antigens, which might stop the immune system from injuring the body’s tissues, as occurs in autoimmune diseases [19].

**Figure 5:**
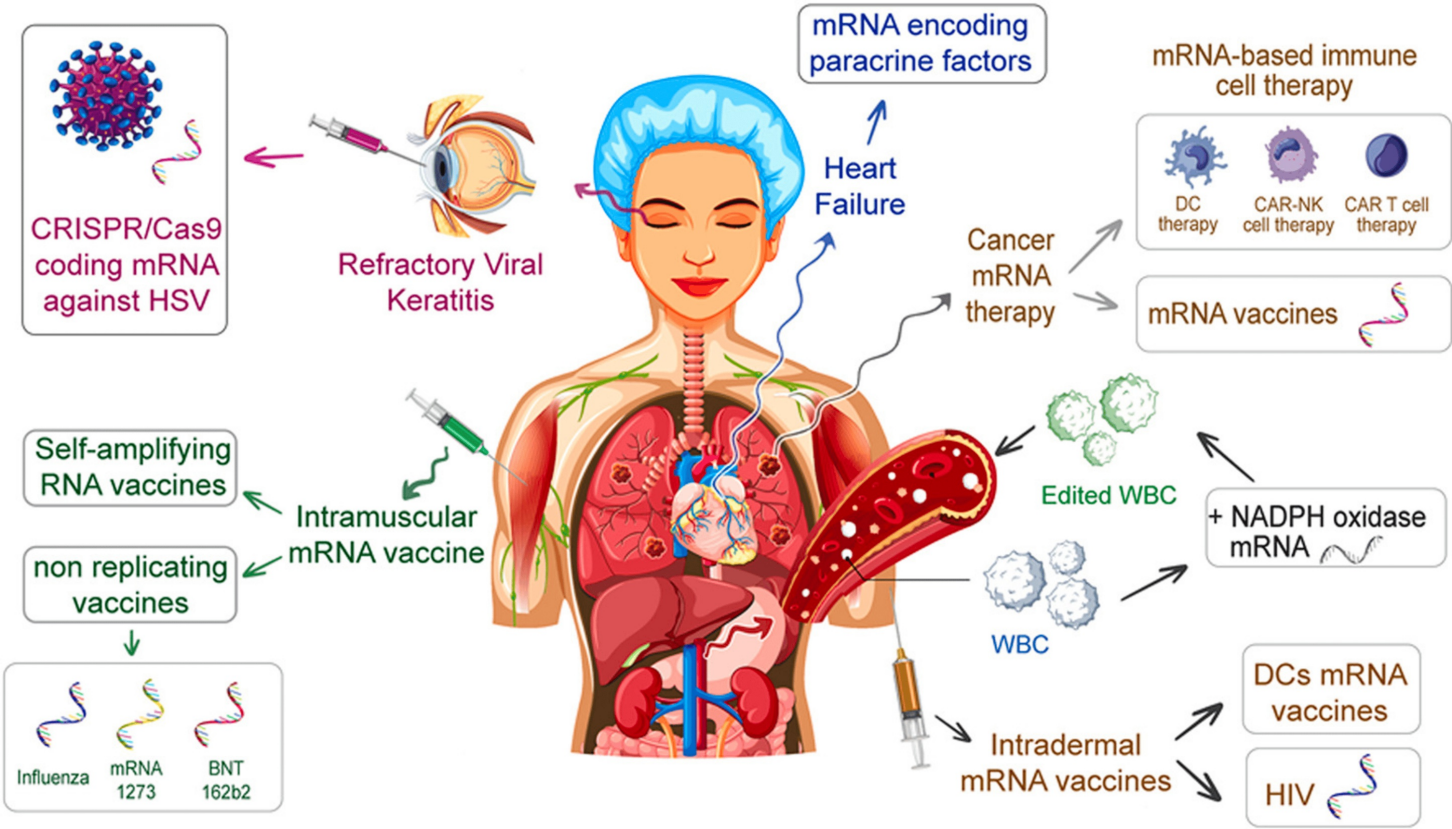
Applications of mRNA Technology in Therapeutics and Vaccines Source: Deyhimfar R, Baradaran B, Baradaran P, et al.: The clinical impact of mRNA therapeutics in the treatment of cancers, infections, genetic disorders, and autoimmune diseases. Heliyon. 2023, 10:10.1016/j.heliyon.2023.e26971 [20]. This figure is licensed under the terms of the Creative Commons CC-BY license. CRISPR: clustered regularly interspaced short palindromic repeats; Cas9: CRISPR-associated protein 9; mRNA: messenger ribonucleic acid; HSV: herpes simplex virus; DC: dendritic cell; CAR-NK: chimeric antigen receptor-natural killer; CAR T: chimeric antigen receptor T cell; NADPH: nicotinamide adenine dinucleotide phosphate; BNT162b2: BioNTech/Pfizer COVID-19 vaccine; mRNA-1273: Moderna COVID-19 vaccine

Figure 5 highlights the assorted possible applications of mRNA technology in both treatments and vaccine development. This research highlights the ability of mRNA for use in self-amplifying vaccines, as well as in non-replicating vaccines, and in addressing a spectrum of diseases, including cancer, HIV, and heart failure. In addition, it mentions inventions such as clustered regularly interspaced short palindromic repeats (CRISPR)/CRISPR-associated protein 9 (CRISPR/Cas9) coding mRNA for treating genetic disorders such as refractory viral keratitis, mRNA-based immune cell therapy, and mRNA vaccines, including BNT162b2 and mRNA-1273 for influenza and COVID-19. Figure 5 thoroughly accents the comprehensive adaptability of mRNA for gene editing, immunotherapy, and the management of chronic diseases.

Chronic Disease Management and the Importance of Therapeutic mRNA Vaccines: Alongside autoimmune diseases, researchers are investigating the application of mRNA technology for the management of chronic diseases, including cardiovascular disease. As an example, work is ongoing to develop mRNA therapies that guide the body to produce proteins that repair damaged heart tissue or promote regeneration in other tissues. Although mRNA-based therapeutics for chronic diseases are still in their infancy, the platform's flexibility and adaptability suggest a bright future for future treatments [21].

Advantages and limitations of mRNA vaccines

Advantages

Rapid Development and Scalability: A key advantage of mRNA vaccines is the speed at which they can be designed and manufactured in response to emerging pathogens. Other vaccines take months or years, if not decades, to grow live viruses or breed viral proteins. mRNA vaccines, in contrast, are developed in the lab by synthesizing an infectious disease virus's genetic material, enabling speedy design, testing, and manufacturing to address newly discovered infectious diseases. The potential for rapid vaccine production of the mRNA platform was especially exemplified by the COVID-19 pandemic when vaccines were engineered in a span of weeks, reflecting their ability to adapt and speed in times of pandemic response [22]. Additionally, scalability is one of the strong points. Since mRNA is being produced synthetically, cell cultures or eggs that are often required in conventional vaccine manufacturing are not needed. This minimizes the risks of contamination and facilitates mass production within relatively short time frames. The capability of rapid expansion in production capacity helps mRNA vaccines respond to global needs, as it did during the COVID-19 pandemic [23].

High Efficacy and Safety Profile: Clinical trial data prove that mRNA vaccines are very effective, especially for COVID-19 vaccination. Phase III trials revealed that the Pfizer-BioNTech (BNT162b2) and Moderna (mRNA-1273) vaccines were more than 90% effective in preventing symptomatic COVID-19 [24]. In addition to efficacy, mRNA vaccines elicit humoral and cellular immune responses, and thus they are a strong weapon against infectious diseases and cancers, and autoimmune disorders. From a safety point of view, mRNA vaccines preclude live viruses, thereby eliminating the risk of vaccine-derived infections associated with conventional live attenuated vaccines. Moreover, because mRNA does not get integrated into the host genome, mutagenesis risk is highly reduced [25].

Flexibility in Designing Vaccines for Different Pathogens: mRNA vaccines are extremely versatile, making it possible to produce vaccines for nearly any pathogen by encoding a target protein of interest. Due to this flexibility, mRNA vaccines can rapidly respond to new strains or virus variants. During the COVID-19 pandemic, companies rapidly changed mRNA vaccines in response to newly emerged variants, illustrating the rapid adaptability of the technology and the possibility of using it for the long term for many infectious diseases [26].

Potential for Personalized Vaccines: One more thrilling benefit of mRNA technology is its potential as personalized cancer vaccines. Tumors tend to have unique mutations, or neoantigens, that can be targeted with mRNA vaccines based on a patient's cancer signature. This means the immune system can identify and destroy cancer cells without harming normal tissue. Personalized mRNA cancer vaccines are in clinical trials today, with early results indicating this technology has the potential to revolutionize cancer treatment [27].

Limitations

Stability Issues (Cold Chain Requirements for Storage): The requirement for ultra-cold storage has limited equitable distribution in resource-limited regions. Innovations such as lyophilized (freeze-dried) formulations or thermostable LNPs are under active investigation to mitigate this barrier. The instability of mRNA and its propensity for degradation mean it must be stored extremely cold to function as designed. The Pfizer-BioNTech COVID-19 vaccine required storage at -70°C (-94°F) at the outset, while the Moderna vaccine needed to be stored at -20°C (-4°F). This creates major logistical problems, especially in countries with low and middle incomes, where the cold chain infrastructure is typically not sufficient [28]. Ongoing analysis looks to create mRNA formulations that are more stable and can withstand higher storage temperatures.

Adverse Reactions and Long-Term Safety Concerns: In clinical trials, mRNA vaccines have demonstrated an encouraging safety record, although many side effects are generally mild to moderate (like injection site pain, fatigue, and headache), a small number of individuals have suffered serious reactions such as anaphylaxis. Although infrequent, these reactions suggest the need for thorough monitoring of adverse reactions that follow immunization [29].

Also of concern is the deficiency of long-term safety data concerning mRNA vaccines. Since this technology is quite new, thorough studies over the long term are necessary to evaluate possible delayed side effects. At this time, no evidence of lasting genetic effects [30]

Production and Distribution Obstacles, Especially in Low-Income Nations: The Scalability of mRNA vaccines exists; however, production is costly because it requires specialized equipment, including lipid nanoparticle (LNP) delivery systems that protect and encapsulate the mRNA. These components are important for delivering mRNA to cells unharmed, but they also complicate the production process. In low-income countries, components of distribution - especially cold chain requirements, cost considerations, and infrastructure constraints - often complicate access to mRNA vaccines. Resolving these issues will require investments in cold chain technology, together with local vaccine production skills, as well as international partnerships to establish equitable access to these crucial vaccines [31].

mRNA vaccine production and future manufacturing innovations

Current Manufacturing Processes

A review of the production process for mRNA vaccines: The fabrication of mRNA vaccines includes a variety of important steps that set it apart from standard vaccine platforms. The identification of target antigens represents the initial requirement because antigens usually stem from viral proteins. Scientists use in vitro methods to synthesize mRNA encoding this antigen after its identification. The mRNA sequence originates from viral genomic material, and scientists have optimized it for human cell translation.

RNA polymerase facilitates the in vitro generation of mRNA from DNA templates as an enzyme. Environmental quality stands as the main objective during a protocol that removes extra nucleotides and DNA templates from synthesized mRNA. The purified mRNA receives protection through lipid nanoparticles (LNPs), which also help the material enter human cells [32].

The Function of Lipid Nanoparticles (LNPs) in Vaccine Distribution: The main operational role of LNPs exists for mRNA vaccines. Human enzymes known as RNases break down mRNA in the body, thus, the use of LNPs becomes necessary. The protective LNPs applied to mRNA create conditions for its survival and delivery to target cells without degradation. The design of LNPs achieves mRNA cellular uptake enhancement through endocytosis promotion, which represents the cellular process of substance intake. Inside the cell, LNPs unlock the mRNA and facilitate the movement to the cytoplasm, so it can synthesize the aimed protein. The lipid nanoparticles contain biodegradable lipids that are in harmony with human cells, thus lessening toxicity and improving efficiency in delivery [28]. Improvements in LNP formulation have markedly enhanced the safety and efficacy of mRNA vaccines, making them an important part of the production process.

**Figure 6:**
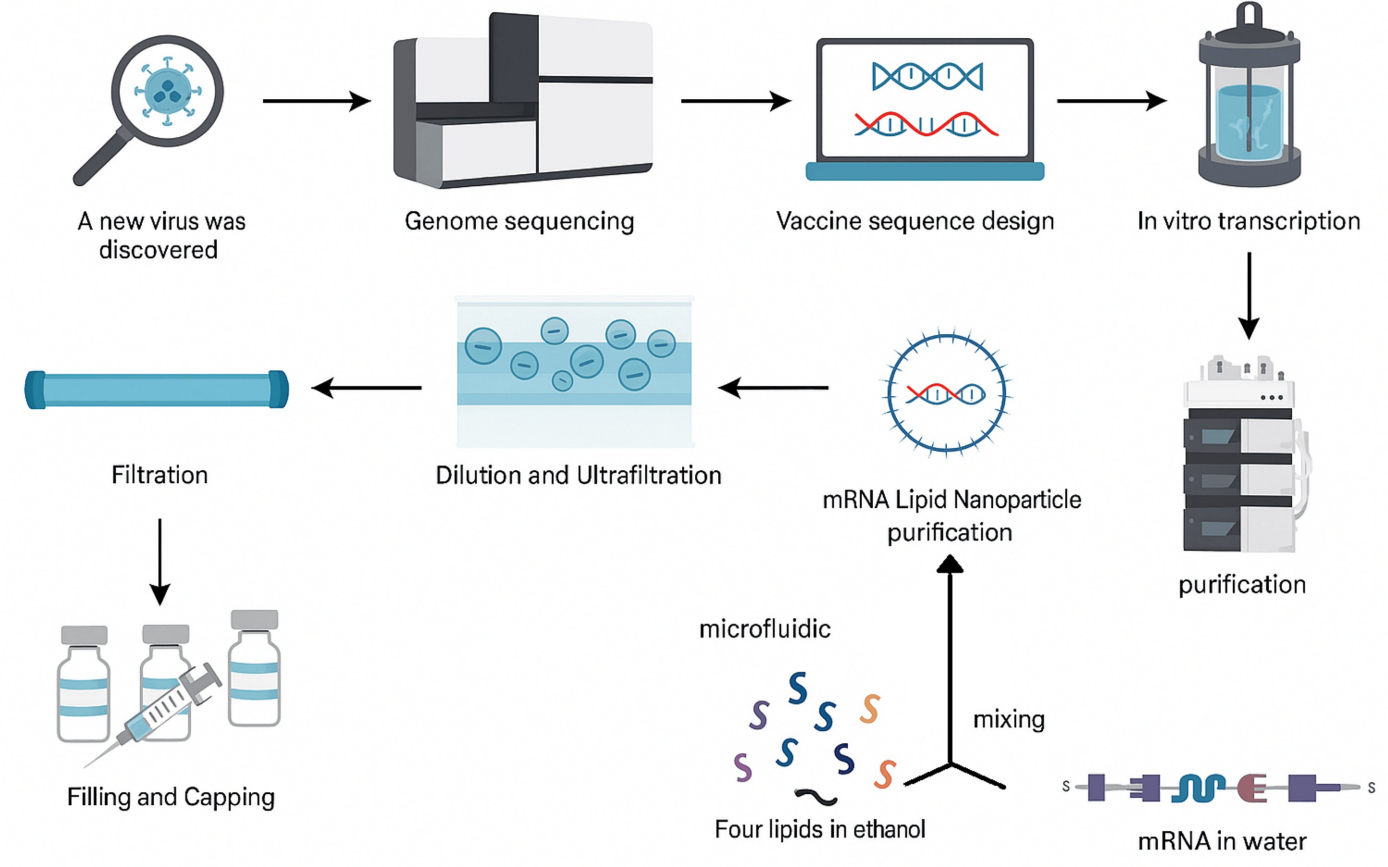
Schematic Overview of mRNA Vaccine Production and Purification Process Source: Fang E, Liu X, Li M, et al. Advances in COVID-19 mRNA vaccine development. Signal Transduct Target Ther. 2022;7(1):94. doi:10.1038/s41392-022-00950-y [33] This figure is licensed under a Creative Commons Attribution 4.0 International License.

Figure 6 demonstrates the mRNA vaccine development process, starting with the discovery of a new virus, followed by genome sequencing to identify the genetic code of the viral protein of interest. Based on this information, scientists design the mRNA sequence that will instruct human cells to produce a harmless version of the viral protein, triggering an immune response. The designed sequence undergoes in vitro transcription, where it is converted into mRNA using enzymatic processes. The transcribed mRNA is then purified to remove unwanted components such as DNA templates, enzymes, and byproducts. Next, the purified mRNA is combined with four types of lipids in ethanol using a microfluidic mixing system to form mRNA lipid nanoparticles, which protect the mRNA and aid its delivery into human cells. This mixture is then diluted and ultrafiltered to remove solvents and adjust the concentration. Further filtration ensures sterility before the vaccine is finally filled into vials and capped, ready for distribution.

Innovations in mRNA Vaccine Production

Progress in Storage Stability along with Temperature Requirements: One of the principal obstacles in the early deployment of mRNA vaccines was their requirement for ultra-low temperature storage. To demonstrate, the Pfizer-BioNTech COVID-19 vaccine needed storage at -70°C, which generated serious issues for distribution, particularly in regions lacking resources [34]. Recent developments in mRNA formulation and stabilization have made it possible to maintain more flexible storage conditions. The Moderna mRNA vaccine, as an example, is engineered to maintain stability at -20°C and may remain stored in regular refrigeration (2-8°C) for up to 30 days [35]. Investigators are working to improve the thermostability of mRNA vaccines by modifying the architecture of mRNA and the chemical makeup of lipid nanoparticles. The aim is to establish formulations that are stable at room temperature, which would greatly ease both distribution and storage, particularly in areas with inadequate cold chain infrastructure.

Economic Methods for Manufacturing and Distributing Products: Scaling production to meet global demand has made the cost of manufacturing mRNA vaccines a major worry. Standard vaccine production technologies typically incorporate difficult biological methods, including the raising of viruses in cell cultures or eggs, whereas mRNA vaccines can result from chemical synthesis, thereby bringing down production time and costs. Improvements in high-throughput RNA synthesis in conjunction with automation and purification strategies have lowered the financial entry points to mRNA vaccine production, enhancing the capability for extensive rollout. Also, decentralized manufacturing models are under investigation to facilitate the placement of production facilities near areas of great demand. This technique can cut transportation costs and increase access in countries with low and middle incomes [36]. Investigations by researchers into new lipid formulations and delivery systems are an important part of their work to boost cost-effectiveness by potentially lowering the need for cold storage, which can decrease distribution costs. The research team investigated dry powder formulations of mRNA vaccines over liquid versions because scientists believe these formulations would offer better stability and transport capabilities.

The Function of Artificial Intelligence and Computational Biology in the Creation of mRNA Vaccines: The development of mRNA vaccines receives increasing benefits from artificial intelligence (AI) and computational biology applications in their design process. Large genetic and proteomic data are analyzed through AI algorithms to identify suitable antigenic targets for vaccine development. The applications enhance mRNA sequences through stability improvement and translation efficiency enhancement, and immunogenicity enhancement. The value of AI has become particularly noticeable in its capability to predict how tiny modifications in the mRNA sequence affect its secondary structure and subsequently affect protein translation efficiency. Researchers can more quickly assess and make changes to vaccine designs, thanks to models that use AI, which markedly lessens the time taken for development. Design is not the only recipient of AI's benefits; it's also supporting manufacturing. Machine learning algorithms analyze production data to show inefficiencies and offer improvements for the synthesis, purification, and formulation processes linked to mRNA vaccine production. Such a situation may produce improved production techniques and could also bring down costs [37].

Ethical and regulatory considerations

Ethical Issues in mRNA Vaccine Deployment

Vaccine Equity - Access in Poor Countries vs. Rich Nations: The principal ethical concern related to mRNA vaccines is the variation in access between countries with high income and nations that are developing. In the context of the COVID-19 pandemic, affluent nations could easily access large stocks of mRNA vaccines, while many countries of low and middle income had a hard time obtaining enough doses. This disparate distribution points out the requirement for international strategies to make certain that life-saving vaccines are reachable to all, without consideration of geographic or economic barriers [38]. Global inequities in vaccine access during the COVID-19 pandemic highlighted the need for more robust international frameworks, such as equitable licensing and regional manufacturing capabilities [16]. COVAX was designed (by Gavi, the Vaccine Alliance, the World Health Organization (WHO), and the Coalition for Epidemic Preparedness Innovations (CEPI), with UNICEF distribution support) to redress global vaccine inequality, but persistent issues related to logistics and inadequate infrastructure are still causing challenges to vaccine distribution in numerous parts of the world.

Ethical considerations emphasize transparent communication of risk-benefit profiles, particularly given the accelerated timelines and novel mechanisms of the mRNA platform. Those involved in trials need to be entirely aware of the risks and benefits associated with the vaccine, especially because of the novel characteristics of mRNA technology. As part of the emergency approval for COVID-19 vaccines, some raised alarms about the quickness of the trials and the possible inadequacy of patient education about long-term safety information. A pledge to ethical best practices demonstrates that clinical trials must follow exacting standards for informed consent while continuing dialogue about trial risks, outcomes, and benefits [39].

Regulatory Challenges

Fast-Track Approval Processes During Pandemics vs. Regular Approval Timelines: To fast-track the deployment of mRNA vaccines, the COVID-19 pandemic created a need for emergency use authorizations (EUAs). Even so, the shortened approval process raises ambiguities about whether a detailed assessment of extensive safety and efficacy data has taken place [40]. Addressing the public crisis requires this strategy.

Global Harmonization of Regulatory Standards for mRNA Vaccines: The major hurdle in mRNA vaccine regulation is the unavailability of globally consistent standards. The U.S. Food and Drug Administration (FDA) and the European Medicines Agency (EMA) require divergent criteria for the approval of vaccines. Due to the rising use of mRNA vaccines, there is a need for international regulatory systems to come into alignment so they can maintain safety, efficacy, and quality in global markets [41].

Future directions and research gaps

Unanswered Questions in mRNA Vaccine Research

Long-range Immune Response and the Durability of Protection: Despite promising short-term efficacy, the duration of immune protection induced by mRNA vaccines, particularly in the context of rapidly mutating viruses or immunocompromised hosts, remains to be fully established. Ongoing longitudinal studies are essential to determine booster requirements and correlate immune markers with protection. The vaccines have shown a strong production of neutralizing antibodies right after administration; however, it is still unclear for how long this protection holds and if booster doses will be essential to maintain immunity. Longitudinal studies are important for figuring out the total immunity time and, particularly, in response to viral mutations and variants that might influence the strength of the initial vaccine [42].

Safety in Special Populations: A second major research topic focuses on making clear the safety and effectiveness of mRNA vaccines in special demographics, such as children, pregnant women, and those who are immunodeficient. During the COVID-19 pandemic, mRNA vaccines have been recommended for some populations, but there is still a deficit of data from large clinical trials that specifically assess their safety in these groups. The lack of representation of pregnancy in clinical trials has caused a careful approach to vaccine guidance for expectant mothers. Just as well, those with compromised immune systems, such as cancer patients throughout treatment and organ transplant patients, may manifest differing responses to mRNA vaccination, requiring additional work to alleviate these safety and efficacy issues [43].

Next-Generation mRNA Vaccines

Study on Self-Amplifying mRNA (saRNA) Vaccines: Self-amplifying mRNA (saRNA) vaccines show great promise in the future as a research area of mRNA vaccine development. saRNA platforms offer dose-sparing advantages and may enable more robust immune responses at lower mRNA quantities. However, challenges in safety, replicon stability, and large-scale Good Manufacturing Practice (GMP) production need to be addressed before clinical translation. Unlike typical mRNA vaccines, which need large quantities of mRNA to provoke an immune response, saRNA vaccines carry additional sequences that permit the mRNA to duplicate itself inside the cell. The intensified amplification of itself may generate greater antigen production from a smaller beginning dose of mRNA, possibly necessitating a smaller vaccination amount and thereby cutting down production costs. First studies conducted in the preclinical phase reveal that saRNA vaccines can produce strong immune responses using fewer doses than conventional mRNA vaccines, which might allow for faster mass vaccination in global health crises [44].

Role of Combination Vaccines: As mRNA technology evolves, the interest in developing combination vaccines that defend against several diseases with one dose is increasing. mRNA vaccines feature a flexible platform that permits the encoding of multiple antigens from assorted pathogens. To illustrate, work is underway to make combination mRNA vaccines that defend against both COVID-19 and influenza. The use of combination vaccines may be especially helpful in decreasing the number of injections needed for safeguarding against different diseases, raising vaccine compliance, and streamlining distribution [45].

Progress in mRNA Vaccines for Gene Editing and Therapeutics for Conditions Other Than Infectious Diseases: Along with their applications in vaccines, researchers are also examining mRNA technologies for possible applications in gene editing and several other therapeutic fields. By allowing the encoding of proteins, mRNA is an optimistic tool for the treatment of genetic disorders that can directly convey therapeutic proteins to cells. Research is happening to produce mRNA therapies for conditions such as cystic fibrosis and particular types of muscular dystrophy. The look into mRNA technology includes its capacity for gene editing, mainly by delivering the CRISPR-Cas9 system through mRNA for precise genomic editing. The introduction of these therapeutic innovations expands the capabilities of mRNA beyond infectious diseases, preparing the ground for new treatments of genetic and chronic conditions [46]. The future of mRNA vaccine technology shows promise, but it still has many unresolved questions. An examination of the sustained immune response and safety of vaccination in special populations, along with innovations such as self-amplifying mRNA and combination vaccines, is important for fully realizing the promise of mRNA vaccines. Research into mRNA for therapeutic purposes beyond infectious diseases may revolutionize treatment for genetic and chronic conditions, introducing a new direction for medicine.

Future Outlook

In the future, the promise of mRNA vaccines applies to conditions outside of infectious diseases. Currently, researchers are hard at work using mRNA technology to construct vaccines and handle problems in cancer, autoimmune diseases, and chronic conditions. In the field of cancer treatment, mRNA vaccines have indicated a potential to generate immune responses directed at tumour-specific antigens, which is opening the avenue for personalized cancer immunotherapy. Inquiries into saRNA and combination vaccines propose the ability to significantly reduce vaccine doses, enable protection from several diseases with a single injection, and thus enhance the practicality and success of large vaccination plans. Research is in progress to examine the application of mRNA vaccines related to autoimmune diseases, together with gene therapy. The realm of mRNA's potential to encode therapeutic proteins is generating a lot of excitement among those who are exploring its application for conditions such as multiple sclerosis and cystic fibrosis, which have the potential to reshape the treatment landscape for genetic disorders and chronic health problems [47].

Importance of Continued Research and Development

For mRNA vaccines to completely fulfil their benefits, ongoing investment in research and development is needed. Unanswered questions remain about the extended immune response, the safety of special populations, and the capability of these vaccines to operate globally. The efficient deployment of mRNA vaccines on a global scale requires us to improve stability for simpler distribution and identify cost-effective manufacturing techniques. Also, for regulators, researchers, and the pharmaceutical field to cooperate is critical to overcome ethical difficulties, such as vaccine equity and the role of informed consent in clinical trials. The success of mRNA vaccines against COVID-19 marks only the first step. Continuing our research efforts could allow mRNA technology to not just deal with pandemics, but also to change the global health landscape by addressing some of the most critical diseases of the moment, including cancer, autoimmune conditions, and newly emerging infectious diseases.

## Conclusions

The efficacy of mRNA vaccines in the COVID-19 pandemic has spurred a paradigm shift in vaccinology, proving the revolutionary power of this technology to fight infectious diseases. mRNA vaccines have many strengths over conventional vaccine platforms, such as speedy development, scalability, and high efficacy. The Pfizer-BioNTech and Moderna vaccines, for instance, had efficacy levels greater than 90% in preventing symptomatic COVID-19 cases, representing a significant breakthrough for global health. Beyond COVID-19, mRNA technology offers a flexible platform that may transform vaccine development for numerous diseases, from emerging infectious diseases to re-emerging pathogens. Its potential for designing vaccines aimed at specific pathogens and individualized medicine, most notably in oncology, presents new opportunities to treat cancer by targeting patient-specific tumor antigens.

In the future, sustained innovation in thermostability, immune longevity, and individualized antigen design will play a pivotal role in realizing the full potential of mRNA vaccines, particularly in the domains of oncology, non-communicable diseases, and during pandemics in the future. International collaboration will also play a key role in addressing issues of ethics, logistics, and regulation, as well as in making these vaccines available equitably and under the same regulatory parameters globally. These initiatives will be instrumental to making sure that mRNA vaccines remain a force to be reckoned with in global health, revolutionizing the way we address diseases today and tomorrow.
